# Feature Extraction Based on Local Histogram with Unequal Bins and a Recurrent Neural Network for the Diagnosis of Kidney Diseases from CT Images

**DOI:** 10.3390/bioengineering11030220

**Published:** 2024-02-25

**Authors:** Abdorreza Alavi Gharahbagh, Vahid Hajihashemi, José J. M. Machado, João Manuel R. S. Tavares

**Affiliations:** 1 Faculdade de Engenharia, Universidade do Porto, Rua Dr. Roberto Frias, s/n, 4200-465 Porto, Portugal; up202003516@edu.fe.up.pt (A.A.G.); hajihashemi.vahid@ieee.org (V.H.); 2 Instituto de Ciência e Inovação em Engenharia Mecânica e Engenharia Industrial, Departamento de Engenharia Mecânica, Faculdade de Engenharia, Universidade do Porto, Rua Dr. Roberto Frias, s/n, 4200-465 Porto, Portugal; jjmm@fe.up.pt

**Keywords:** kidney cancer, medical imaging, image analysis, machine learning, local statistical distribution

## Abstract

Kidney disease remains one of the most common ailments worldwide, with cancer being one of its most common forms. Early diagnosis can significantly increase the good prognosis for the patient. The development of an artificial intelligence-based system to assist in kidney cancer diagnosis is crucial because kidney illness is a global health concern, and there are limited nephrologists qualified to evaluate kidney cancer. Diagnosing and categorising different forms of renal failure presents the biggest treatment hurdle for kidney cancer. Thus, this article presents a novel method for detecting and classifying kidney cancer subgroups in Computed Tomography (CT) images based on an asymmetric local statistical pixel distribution. In the first step, the input image is non-overlapping windowed, and a statistical distribution of its pixels in each cancer type is built. Then, the method builds the asymmetric statistical distribution of the image’s gradient pixels. Finally, the cancer type is identified by applying the two built statistical distributions to a Deep Neural Network (DNN). The proposed method was evaluated using a dataset collected and authorised by the Dhaka Central International Medical Hospital in Bangladesh, which includes 12,446 CT images of the whole abdomen and urogram, acquired with and without contrast. Based on the results, it is possible to confirm that the proposed method outperformed state-of-the-art methods in terms of the usual correctness criteria. The accuracy of the proposed method for all kidney cancer subtypes presented in the dataset was 99.89%, which is promising.

## 1. Introduction

Two kidneys are in the human body, located behind the abdomen and protected by the thorax. Blood purification is the most critical function of the kidneys. To keep the blood and body cells clean, the kidneys remove toxic substances produced during metabolic processes in the blood. In addition, the kidneys play a fundamental role in vitamin D metabolism [1,2]. Kidney failure can lead to death within a few days if the blood is not cleansed. Despite all the advances, kidney cancer continues to spread worldwide, with over 400,000 new cases diagnosed each year [1]. Unlike most cancers, 59% of the kidney cancer cases occur in developed countries, and the mortality rate of kidney cancer is higher in these countries [1,2]. More than 10% of the world’s population suffers from Chronic Kidney Disease (CKD), which was ranked as the 16th leading cause of death in 2016 and is expected to rise to 5th place by 2040. Kidney cancer is the ninth most common cancer in men and the fourteenth most common cancer in women [3,4]. There is a severe shortage of nephrologists and radiologists in many regions of the world. For example, in South Asia, the ratio of nephrologists to the population is extremely low, with only one nephrologist per one million people. Kidney cancer usually manifests as nodules; although, tumours can form in one or both kidneys. Malignant cells can enter the bloodstream and affect other organs [5,6]. Approximately 90% of the kidney cancers are caused by metastasis. Cyst formation, nephrolithiasis, or kidney stones, and renal cell carcinoma, i.e., kidney tumours, are the most common diseases affecting kidney function after metastasis. Early diagnosis of kidney disorders such as cysts, stones, and tumours is essential to prevent kidney failure. The prevalence of kidney disease, the global shortage of nephrologists and radiologists, and the advent of medical systems based on Deep Learning (DL) paradigms highlight the importance of developing Artificial Intelligence (AI) solutions to diagnose kidney cancer from medical images. Such solutions can help physicians and reduce patient distress [7]. Many recent studies have been conducted to diagnose kidney cancer using medical images. The limitations of these methods include the lack of valid datasets, the diversity of the medical imaging devices, and the structural differences between images acquired using different devices. Compared with long-established Machine Learning (ML) methods, DL methods have led to the development of new medical systems. Concepts such as pre-trained DL models have also been considered in diagnosing kidney cancer abnormalities. Based on these explanations, a DL-based method for kidney cancer classification is proposed in this article. The main innovations of the proposed method are as follows:

- The use of gradients and histograms with asymmetric intervals to extract features to classify kidney cancer subtypes accurately;
- The use of feature extraction before applying the DL model to reduce the dimensionality of the input data compared to the conventional methods;
- The reduction in the dimensions of the input data to increase the training speed and reduce the complexity of the used DL model.

The proposed method, which involves calculating the intensity and gradient histograms of the input kidney Computed Tomography (CT) image at asymmetric intervals, reduces the input dimensions and effectively diagnoses various kidney cancer-related complications. The structure of the remainder of this article is as follows: The following section provides background information and details of applying ML and DL techniques to detect kidney abnormalities. Section 3 explains the methodology and the proposed method. Section 4 describes the obtained results and compares the proposed method with recent studies in this field. Finally, Section 5 provides the conclusion.

## 2. Literature Review

ML-based methods can accurately segment healthy patients or those with small nodes. However, kidney segmentation is particularly challenging [8,9]. Verma et al. [10] preprocessed kidney ultrasound images using median and Gaussian filters and morphological operations. Using Principal Component Analysis (PCA), the authors extracted image information and classified the kidney images using the K-nearest Neighbour (KNN) algorithm. Khalifa et al. [11] presented a 3D technique for characterising kidneys in abdominal CT images using a level set-based deformable model and Markov–Gibbs random field. Wolz et al. [12] proposed an approach based on a hierarchical atlas registration and weighting scheme to characterise different body organs in abdominal CT images. Yang et al. [13] also proposed a two-step method for kidney segmentation in CT angiographic images based on multi-atlas image registration. Zhao et al. [14] presented a 3D approach to segment kidneys in 3D CT images, which employed an iterative technique to improve resolution. Shehata et al. [15] employed a level set and Markov–Gibbs random field-based framework to discriminate kidneys in diffusion Magnetic Resonance (MR) images. Khalifa et al. [16] used a composite framework that combined non-negative matrix factorisation with a guided active contour model to segment kidneys in 3D images. Skalski et al. [17] proposed a dynamic contour-based method using a level-set structure for kidney segmentation in CT images.

Several ML algorithms have been investigated for kidney image classification, including Decision Trees (DT), Random Forest (RF), Support Vector Machines (SVM), Multilayer Perceptron (MLP), Naive Bayes, and KNN [18]. The best results were obtained using the KNN and Naive Bayes classifiers. Due to the increasing use of DL in image processing and classification, many studies have been conducted to explore its applications. One of the most promising DL applications can be found in the field of medical image processing and analysis. For example, transfer-learning techniques have been used to process medical images using pre-trained DL models. Compared with conventional DL models, pre-trained models have shown better results. ResNet [19], InceptionNet [20], ExceptionNet [21], and EfficientNet [22] are some of the pre-trained models that use transfer learning in medical image classification. Many studies have used attention-based deep pre-trained models for image analysis, including Vision Transformer (ViT) [23], Big Transformer (BiT) [24], External Attention Network (EANet) [25], Compact Convolutional Transformer (CCT) [26], and Shifted Window Transformer (SWT) [27]. Therefore, the number of DL approaches that have been proposed to assist the diagnosis of diseases from image data is continuously increasing. Yang et al. [28], Haghighi et al. [29], and Mehta et al. [30] used fully connected convolutional networks to segment kidney images. Fu et al. [31] used ultrasound images to input an attention module to segment kidney cysts in CT images. Da Cruz et al. [32] presented a fully automated method for segmenting kidneys with and without tumours on CT images. The method consisted of a preprocessing step for histogram normalisation, CNN models, and a post-processing block. The first CNN model for CT image classification and feature reduction was AlexNet, whereas the second model was U-Net, which accurately segmented the kidneys. In [18,33], kidney ultrasound images were analysed using pre-trained DNN models, mainly ResNet-101, ShuffleNet, and MobileNet-v2, for feature extraction, and an SVM, for the classification. The above review indicates that research on kidney images can be divided into two main categories: one focuses on kidney image segmentation, and the other focuses on diagnosing kidney disorders. Due to the limited number of studies addressing the diagnosis of kidney disease using medical images and the complexity of the required processing, this study proposes a new method to identify and categorise kidney cancer subtypes in CT images.

## 3. Materials and Methods

**Figure 1:**
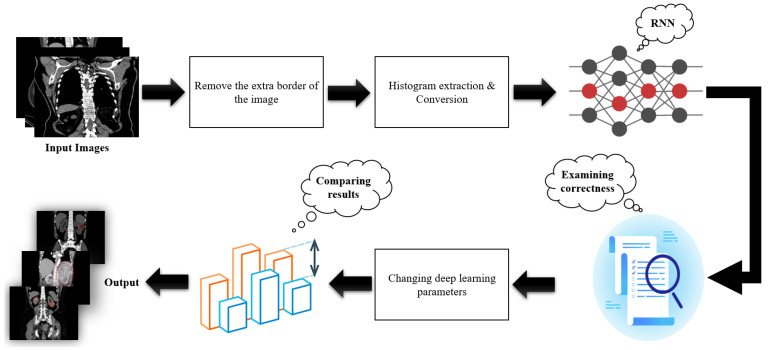
Diagram of the developed methodology.

In this section, the theoretical details of the proposed method are given, along with the selection of its parameter values. Figure 1 shows the block diagram of the adopted methodology. Removing extra borders of the input image is a preprocessing step. The main steps of the methodology include the extraction of the input image histogram and the use of a Recurrent Neural Network (RNN). The selection of the correct asymmetric intervals in the histogram extraction and the use of the RNN model are novelties of the present study. Two blocks, namely for examining correctness and adjusting the DL parameters, were added to maximise the efficiency of the developed methodology. The final step involves the comparison and validation of the obtained results.

### 3.1. Image Black Border Removal

**Figure 2:**
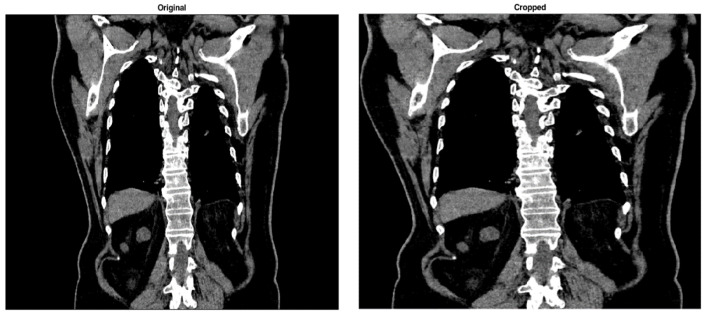
A CT image before (on the **left**) and after removing its black margin (on the **right**).

The border-cutting step seeks to expedite the further feature extraction by shrinking the input image and eliminating extraneous details. Figure 2 shows an original CT image and the corresponding one obtained after applying this procedure. With this processing, the dark margin of the input image is eliminated, and the image is reduced. Furthermore, because CT images contain many pixels with nearly 0 (zero) intensity values, removing these margin pixels decreases the severity of the histogram imbalance.

### 3.2. Local Histogram with Asymmetric Intervals

**Figure 3:**
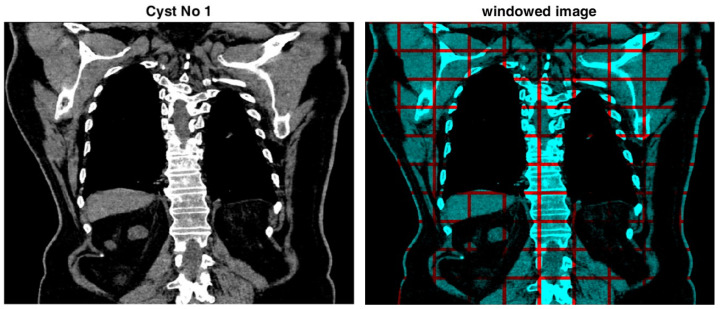
An original CT image (on the **left**) and the corresponding windowed image (on the **right**).

An asymmetric interval is taken onto the reference to find the normalised histogram of the regions in the cropped image. The reason for choosing an asymmetric interval for the histogram is the higher pixel density in the darker intervals of the input image compared with the lighter ones. This asymmetry is necessary to achieve an adequate separation in the statistical representation of the image’s pixels. A well-known weakness of the histogram is its global form. To solve this issue, windowing is performed, which involves dividing the input CT image into 100 windows with identical dimensions, i.e., ten intervals in width and height. An asymmetric normalised histogram is then built independently for each window. An eight-point histogram yields a 100×8 feature matrix for the input image. Figure 3 shows an example of a windowed image.

### 3.3. Image Gradient Histogram with Asymmetric Intervals

In addition to extracting the input CT image’s local histogram, the image’s gradient histogram is used to detect the edges associated with the kidney. As all kidney complications are usually visible as edges, the density of these edges can provide a statistical expression of the kidney condition. Similar to the previous step, the input image is first divided into 100 windows with equal dimensions, and then, the asymmetric normalised histogram is built from its gradient. The intervals of the gradient histogram are chosen differently from those of the intensity image because the gradient has a much higher density in the dark regions of the input image; therefore, the intervals are much denser in the 0 regions and more sparse in the lighter areas of the image. The resulting 100×8 matrix is merged with the intensity histogram, and the merged matrix is converted into a vector and used as a 1×1600 input for a DNN.

### 3.4. Recurrent Neural Network

**Figure 4:**
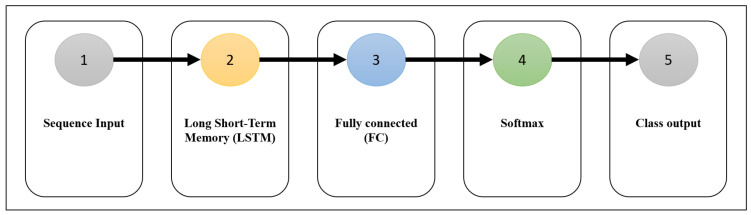
The architecture of the used RNN.

Figure 4 illustrates the architecture of the RNN used in this study, which consists of sequential input and output layers and three additional layers. The second layer is a Long Short-Term Memory Network (LSTM), a vital processing layer commonly employed in DL models for sequential data processing.

**Figure 5:**
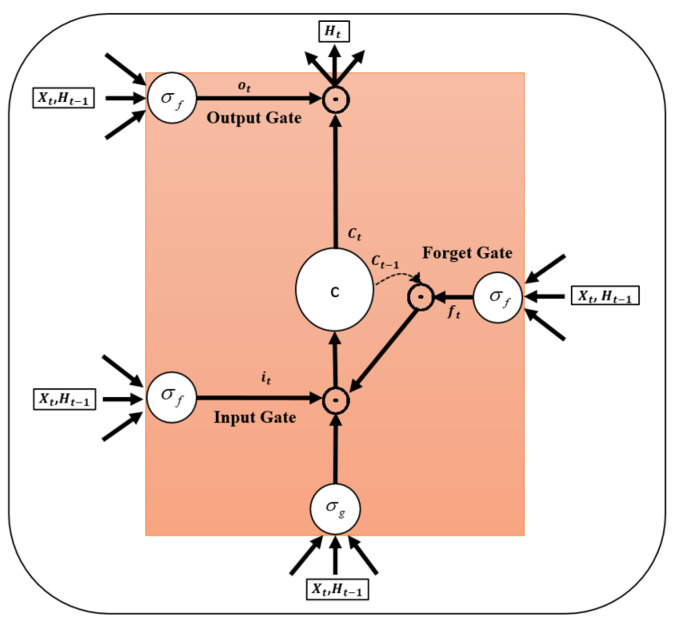
A common LSTM structure.

The LSTM layer can retain sequential input data over long periods, making it useful for various signal processing problems, including of medical imaging. The main advantage of the LSTM layer is that it overcomes the usual memory limitations of the RNNs. In conventional RNNs, the influence of distant samples becomes insignificant and eventually tends toward 0, making them inapplicable for learning long-term dependencies. The LSTM layer solves this problem by updating its weights using gradients while retaining the input data longer. As shown in Figure 5, LSTMs have three main gates, input, forget, and output, which control their output.

Each hidden unit maintains an internal state updated at each time step based on the input and previous state. Increasing the number of hidden units improves the model’s ability to capture complex patterns in the data but also increases the model’s computational complexity and training time. Typically, a combination of LSTM and dropout layers is used in the training phase to prevent overfitting. In the dropout layer, some units of the LSTM layer are randomly disabled during training, which means that they are not updated. However, during the prediction, all units are active. This approach effectively reduces the risk of overfitting. The LSTM layer can operate according to two output modes: sequence mode and last mode. In the first mode, the LSTM generates an output equal to the input length for each input sequence. This mode is often used for time series classification or forecasting tasks. In the second mode, the LSTM produces only one output for the entire input sequence, meaning that only the final response is provided. In addition, an LSTM has several other parameters that control its behaviour, including the gate activation function, weight initialisation, and weight regularisation. Overall, an LSTM is a robust layer for DL models. By incorporating memory cells and information flow-controlling gates, the LSTM layer can effectively capture long-term dependencies in the data while mitigating the challenge of minor sample effects common in traditional RNNs. The mathematical relationships of the LSTM structure shown in Figure 5 are as follows:

(1) $$\begin{array}{c} f_{t}=\sigma_{f}\left(W_{xf}x_{t}+W_{hf}h_{t-1}+b_{f}\right),\\ g_{t}=\sigma_{g}\left(W_{xc}x_{t}+W_{hc}h_{t-1}+b_{c}\right),\\ i_{t}=\sigma_{f}\left(W_{xi}x_{t}+W_{hi}h_{t-1}+b_{i}\right),\\ c_{t}=f_{t}⊙c_{t-1}⊙g_{t}⊙i_{t},\\ o_{t}=\sigma_{f}\left(W_{xo}x_{t}+W_{ho}h_{t-1}+b_{o}\right),\\ h_{t}=o_{t}⊙c_{t}, \end{array}$$

where $x_{t}$ is the input, $h_{t-1}$ is the previous state, $c_{t}$ is the last state saved in the forget gate, *W* is the weight matrix, *b* is the bias of each gate, σ is the activation function, ⊙ is the pointwise multiplication, and $h_{t}$ is the final output. The third layer, the dense layer, is a fully connected layer commonly used in DL models for classification and regression tasks. This layer connects each neuron in the LSTM layer to all neurons in the current layer. The fully connected layer can effectively learn the relationships between the input data and output classes by making such connections. Moreover, the weight matrix is updated during training to minimise errors. In general, the result of the fully connected layer is fed into the output layer through the softmax activation function. This function maps the output to a range of real numbers between 0 (zero) and 1 (one) and creates a probability distribution for the output classes. The softmax activation function is commonly used in DL models involving classification tasks. The output of the softmax layer takes the form of a probability vector, where each element indicates the probability of occurrence of a particular class. The softmax output is calculated by taking the exponential of each input vector element and then normalising the resulting values. This normalisation step ensures that the resultant vector is a probability distribution, a vital characteristic of the softmax activation function. The softmax function is expressed as follows:

(2) $$\mathrm{softmax}\left(x_{i}\right)=\exp\left(x_{i}\right)/sum\left(\exp\left(x_{j}\right)\right),$$

where $x_{i}$ is the input of the ith neuron, and exp is the exponential function. Throughout the training phase, the weights of the LSTM and FC layers are modified to reduce the cross-entropy loss function. The cross-entropy loss function models the dissimilarity between predicted and accurate data. The cross-entropy loss function is described as follows:

(3) $$L=-sum(y_{true}\;⊙y_{pred}),$$

where $y_{true}$ is the actual value, and $y_{pred}$ is the predicted value.

## 4. Results and Discussion

This section presents the used evaluation criteria and dataset, the obtained results, and the comparison of the proposed method with state-of-the-art methods. Thus, first, the evaluation criteria used for the medical image classification methods are presented. Then, the chosen dataset is described. Finally, implementation details, a study on the effects of each method’s parameter on its accuracy, and a comparison between the results of the proposed model and the latest methods in this field are given. All implementations were performed using the MATLAB 2022b software, and the hardware used consisted of an i7-8850H 2.60 GHz (12 CPUs), 16 GB RAM, and an Nvidia Quadro P2000 graphics card.

### 4.1. Evaluation Metrics

The evaluation of classification models usually relies on metrics such as accuracy, precision, recall, F1 score, and the Matthews Correlation Coefficient (MCC). Among these metrics, accuracy, the most commonly used metric, denotes the proportion of correctly classified samples to the total number of samples. The F1 score measures the balance between precision and recall, as it is the harmonic mean of these two metrics. MCC is a valuable metric when there is an imbalance in the number of classes in the used dataset, such as detecting kidney cysts, where there may be many images without cysts compared to those with cysts. MCC simultaneously considers true and false results in positive and negative samples, allowing for a more accurate performance assessment than accuracy or precision alone. Here, True Positive (TP) is defined as a correctly identified sample belonging to a particular class, whereas True Negative (TN) represents a correctly identified sample that does not belong to the corrected class. On the other hand, False Positive (FP) refers to a sample assigned to a class that does not belong, and False Negative (FN) refers to a sample that belongs to a class but is incorrectly identified as not belonging to one [34,35]. Therefore, one has the following evaluation metrics:

(4) $$\begin{array}{c} \mathrm{Accuracy}=\frac{\left(\mathrm{TP}+\mathrm{TN}\right)}{\left(\mathrm{TP}+\mathrm{TN}+\mathrm{FP}+\mathrm{FN}\right)},\\ \mathrm{Precision}=\frac{\mathrm{TP}}{\left(\mathrm{TP}+\mathrm{FP}\right)},\\ \mathrm{Recall}=\frac{\mathrm{TP}}{\left(\mathrm{TP}+\mathrm{FN}\right)},\\ F1\;\mathrm{score}=2\times \left(\mathrm{Precision}\times \mathrm{Recall}\right)/\left(\mathrm{Precision}+\mathrm{Recall}\right),\\ \mathrm{MCC}=\frac{\left(\mathrm{TP}\times \mathrm{TN}-\mathrm{FP}\times \mathrm{FN}\right)}{\sqrt{\left(\mathrm{TP}+\mathrm{FP}\right)\times \left(\mathrm{TP}+\mathrm{FN}\right)\times \left(\mathrm{TN}+\mathrm{FP}\right)\times \left(\mathrm{TN}+\mathrm{FN}\right)}}. \end{array}$$

Additionally, the confusion matrix was used, which is a table containing the values of TP, TN, FP, and FN from which all evaluation criteria can be derived.

### 4.2. Used Dataset

**Figure 6:**
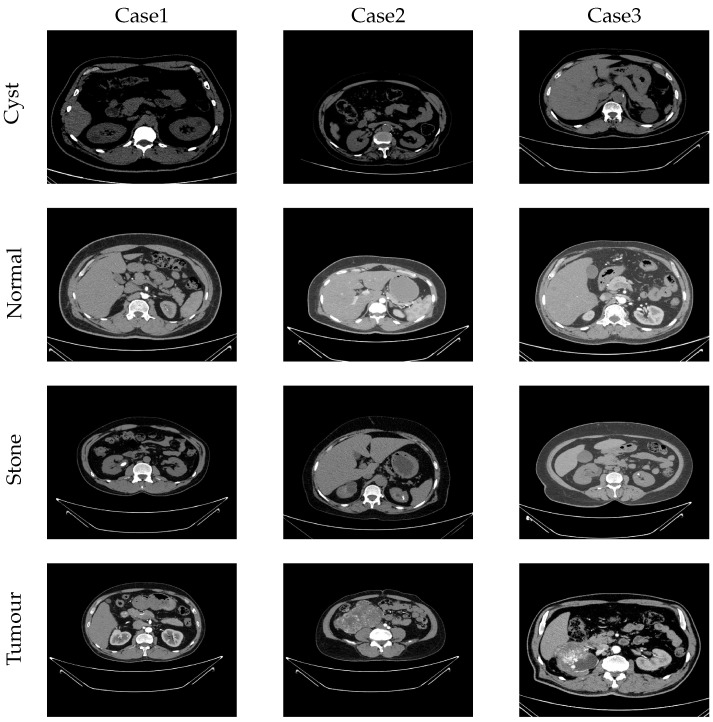
Examples of the images included in the used dataset (delineations in red indicate disease issues) [34].

In this study, a dataset of kidney cysts, stones, and tumours [34] was used, which includes images of healthy, cystic, stone, and tumour-affected kidneys obtained from the Picture Archiving and Communication System (PACS) and workstations of the Dhaka Central International Medical Hospital (DCIMCH), in Dhaka, Bangladesh, where the related data collection and experimental procedures were authorised [34]. According to the dataset description, the image headers, including patient information and various labels, were removed, and the DICOM images were converted to the JPG format. The Philips IntelliSpace Portal software, a standard tool for radiology equipment, was used to label the images. In addition, the DICOM images were converted to JPG format using the Sante DICOM Editor software. Finally, all images were reviewed and labelled by a physician [34]. The used dataset includes 12,446 images, Table 1. Examples of the images included in the dataset are shown in Figure 6, where the red delineations indicate disease issues. For this study, both coronal and axial sections from the CT images of the whole abdomen and urogram, acquired with and without contrast, were selected. The unequal number of samples across categories within the kidney dataset (Table 1) may affect the effectiveness of the method under study. To mitigate this effect on the learning process of the proposed method, approximately 70% (1000 samples) of the smallest category, namely the stone category, was used for training, while the remaining samples were allocated for testing. In addition, an equivalent number of data samples from the other categories was selected for training, which was equal to the training samples from the stone category. This ensured that exactly 1000 samples from each class were used in the training phase, while the remaining samples were assigned for testing (Table 1). Therefore, the used training set included 4000 samples, while 8446 samples were assigned to the test, which means that 32% of the samples were assigned to the training and the rest to the test. Both training and test data were randomly selected.

### 4.3. Experimental Results

This section presents the results obtained in each step of the proposed method and the selection scheme of its critical parameters. The results are discussed taking into account the effect of the CT image intensity histogram intervals on the method’s accuracy, the impact of the histogram of the image gradient intervals on the method’s accuracy, and the performance of state-of-the-art methods.

#### 4.3.1. Selection of Asymmetric Intervals of the Intensity Histogram

**Figure 7:**
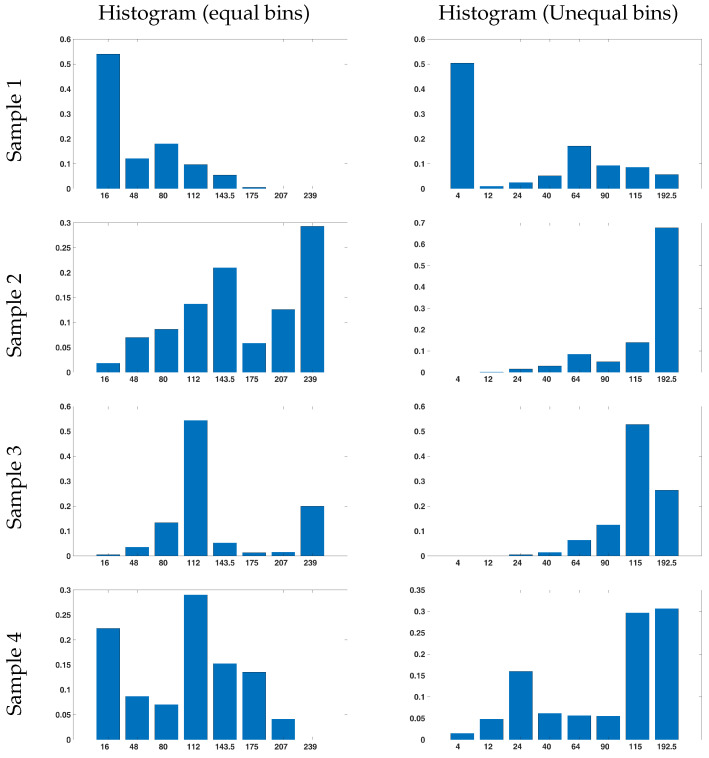
Intensity histograms of a sample window in a CT image in four classes with symmetric intervals (on the **left**) and suggested intervals (on the **right**).

The proposed method builds an asymmetric histogram using different image windows, which is one of the most important contributions of the current study. The asymmetric interval of the histogram is used to separate the pixels with values near 0, owing to the large number of dark pixels in the input image. This approach provides a more accurate statistical representation of CT images. However, the selection of the most suitable asymmetric interval is crucial for obtaining accurate results. The optimal interval should maximise the intraclass similarity of all windows and minimise the similarity of windows in different classes. The average correlation coefficient between each window and its corresponding window in the same classes, less the average correlation coefficient between each window and its corresponding window in other classes, was used to generate an objective function. The chosen histogram interval is assumed to be the same for all the windows. Thus, the input vector of the cost function consists of seven integers between 0 and 255 values without repetition. A genetic algorithm was used to optimise the cost function. The defined optimal intervals are presented in Table 2. As an example, Figure 7 shows four histograms of a window in four different classes in two symmetric and asymmetric histograms. Based on this figure, one can perceive that, in the conventional histogram, the values of the last three intervals are almost 0. However, all of our defined intervals have values showing that the selected intervals have increased histogram data. In addition, the proposed intervals decrease the similarity of the histogram values in different classes, e.g., two, three, and four samples.

#### 4.3.2. Selection of Asymmetric Intervals for the Gradient Histogram

Another important novelty of the proposed method is the building of the asymmetric gradient histogram of the input CT image. The asymmetric intervals of the histogram for feature extraction from the gradient images are different from the used asymmetric intervals of the CT intensity images because they have more dark pixels. This helps distinguish the high density of pixels with near 0 values and improves the feature dissimilarity in different classes. The process for determining the optimal interval is the same as that used for the intensity images, except that the input is derived from the gradient of the input image. Because the gradient captures only edges, it is generally darker than a standard image, which results in a narrower range near 0 values and a broader range in bright areas, as expected. The defined optimal intervals are presented in Table 3.

**Figure 8:**
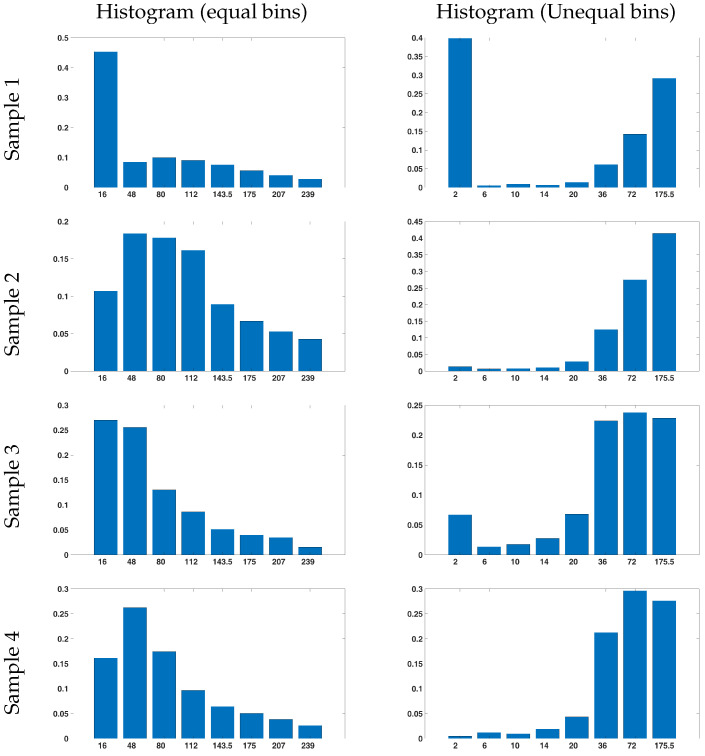
Gradient histograms of a sample window in a CT image with four classes with symmetric intervals (on the **left**) and suggested intervals (on the **right**).

Compared with Table 2, the pixels are dense around 0 values and are widely distributed at higher intervals. To illustrate this, Figure 8 shows four gradient histogram examples in two standard and suggested intervals in a window with four different classes.

### 4.4. Experimental Results

The efficiency of the proposed method was evaluated by training its DL model using four different approaches. First, the histogram features of the CT intensity images with the suggested intervals were used to train the model. Second, the model was trained using the histogram of the CT gradient images. Third, the model was trained using both features. To analyse the effect of asymmetric intervals on the method’s accuracy, a histogram of symmetric intervals was used for feature extraction in both CT intensity and gradient images, and the extracted features were used to train the model. Table 4, Table 5, Table 6 and Table 7 show the confusion matrix built for each of the four classes for the training and test groups. The results confirm that combining the intensity and gradient histograms of the CT images yielded the best accuracy. Table 7 reveals a significant loss of efficiency when standard histograms were used, highlighting the importance of selecting an asymmetric interval for the histogram building.

Table 8 includes state-of-the-art studies that used the same kidney dataset and their corresponding accuracies for each class. In [34,36,37], a better performance than other studies in terms of overall accuracy was found. However, the proposed method outperformed all previous approaches on all correctness criteria. Moreover, regarding the data assigned for training and testing, the proposed method achieved a better response using less training data, indicating its proper efficiency. The proposed method has several unique features that distinguish it from the other methods. One such feature is the use of intensity and gradient histogram features. This reduces the input dimensions and converts the two-dimensional (2D) DL model into a one-dimensional (1D) model. As an advantage, the dimension reduction of input features and conversion to a 1D model reduces the processing and complexity of the model. The second advantage is the feature extraction speed of the proposed method because both the gradient and histogram operators used in the proposed method are computationally fast. Therefore, the proposed method is excellent regarding response time. Based on these findings, one can conclude that the use of histogram and gradient operators in local form can be a simple but effective approach for the classification of medical images in numerous applications.

## 5. Conclusions

In this study, An innovative method for categorising kidney anomalies, including cysts, healthy kidneys, tumours, and stones, was presented. The method uses CT scans of the kidney as input and builds the images’ intensity and gradient histograms from non-overlapping windows. Then, a five-layer DL model featuring an LSTM layer is trained using these attributes to classify CT images according to the kidney issue. The feature extraction step involves the building of the histograms using asymmetric intervals because the CT images and their gradients usually have many pixels with dark values. The optimum selection of asymmetric histogram intervals is the primary factor contributing to the suggested method’s effectiveness. The histogram intervals that maximised the similarity of the retrieved features across classes and the differences in features between classes were chosen using a cost function. Additionally, the resultant features are combined into a feature vector, which significantly reduces the number of weights and the overall complexity of the used DNN model compared to the related two-dimensional model. Compared to current efforts in this field, testing the suggested method under various situations on the chosen dataset showed that it is highly efficient. The method has demonstrated superior performance compared to existing methods, outperforming them in various evaluation criteria. Specifically, it achieved a precision of 0.984, a recall of 0.996, an F1 score of 0.991, and an MCC of 0.990. The effectiveness of the proposed method can be attributed to its distinctive characteristics, such as the thoughtful selection of relevant features and the design of its DL model comprising five layers. The suggested model structure seamlessly aligns with the identified features, which increases the method’s strength and efficiency. As part of future work, an enhanced version of the proposed method could incorporate additional features, such as texture and time-frequency features. This modification will extend the classification capabilities beyond those offered by the initially proposed method, allowing for the identification of a broader range of classes. Validating the proposed method using different datasets is critical to confirm its performance, mainly in clinical usage, and should be addressed in the near future. Moreover, the explainability of the proposed method should be studied to facilitate its use by clinicians.

## Figures and Tables

**Table 1:** Number of images of each class and number of training and testing samples.

	Total	Train	Test
Cyst	3709	1000	2709
Normal	5077	1000	4077
Stone	1377	1000	377
Tumour	2283	1000	1283
Total	12,446	4000	8446

**Table 2:** Normal intervals and suggested intervals for a CT image intensity histogram.

Symmetric Interval	0	32	64	96	128	159	191	223	255
Suggested Range	0	8	16	32	48	80	100	130	255

**Table 3:** Normal intervals and suggested intervals for histogram of the used CT gradient images.

Symmetric Interval	0	32	64	96	128	159	191	223	255
Suggested Range	0	4	8	12	16	24	48	96	255

**Table 4:** Confusion matrix of the proposed method for training and test sets with its model trained with an asymmetric intensity histogram.

	Train				Test			
	**Cyst**	**Normal**	**Stone**	**Tumour**	**Cyst**	**Normal**	**Stone**	**Tumour**
Cyst	1000	0	0	0	2709	0	0	0
Normal	2	998	0	0	3	4074	0	0
Stone	0	2	998	0	0	9	368	0
Tumour	0	0	7	993	0	0	13	1271

**Table 5:** Confusion matrix of the proposed method in the training and test data with its model trained with an asymmetric gradient histogram.

	Train				Test			
	**Cyst**	**Normal**	**Stone**	**Tumour**	**Cyst**	**Normal**	**Stone**	**Tumour**
Cyst	1000	0	0	0	2707	0	0	2
Normal	12	945	0	43	12	4013	19	33
Stone	0	9	991	0	0	17	360	0
Tumour	0	0	8	992	0	0	12	1271

**Table 6:** Confusion matrix of the proposed method trained with combined intensity and gradient histograms.

	Train				Test			
	**Cyst**	**Normal**	**Stone**	**Tumour**	**Cyst**	**Normal**	**Stone**	**Tumour**
Cyst	1000	0	0	0	2709	0	0	0
Normal	2	998	0	0	2	4074	1	0
Stone	0	3	997	0	0	1	360	0
Tumour	0	0	3	997	0	0	5	1278

**Table 7:** Confusion matrix of the proposed method trained with symmetric combined intensity and gradient histograms.

	Train				Test			
	**Cyst**	**Normal**	**Stone**	**Tumour**	**Cyst**	**Normal**	**Stone**	**Tumour**
Cyst	808	0	192	0	2131	0	578	0
Normal	1	989	10	0	0	4066	11	0
Stone	2	13	985	0	0	6	371	0
Tumour	9	1	22	968	22	15	59	1187

**Table 8:** Comparison of the proposed and latest related methods.

Model	Accuracy	Class	Precision	Recall	F1 Score	MCC
YOLOv7 [35]	—	Cyst	0.892	0.633	0.74	0.673
		Normal	—	—	—	—
		Stone	0.819	0.855	0.836	0.816
		Tumour	0.936	1	0.966	0.960
		Average	0.882	0.829	0.854	0.648
EANet [34]	77.02%	Cyst	0.593	1	0.745	0.788
		Normal	0.896	0.848	0.871	0.616
		Stone	0.845	0.495	0.624	0.821
		Tumour	0.93	0.777	0.847	0.994
Swin Transformer [34]	99.30%	Cyst	0.996	0.996	0.996	0.981
		Normal	0.996	0.981	0.988	0.983
		Stone	0.981	0.989	0.985	0.996
		Tumour	0.993	1	0.996	0.923
CCT [34]	96.54%	Cyst	0.968	0.923	0.945	0.970
		Normal	0.989	0.975	0.982	0.966
		Stone	0.94	1	0.969	0.956
		Tumour	0.964	0.964	0.964	0.974
VGG16 [34]	98.20%	Cyst	0.996	0.968	0.982	0.965
		Normal	0.985	0.973	0.979	0.974
		Stone	0.966	0.988	0.977	0.986
		Tumour	0.982	0.996	0.989	0.596
Inception v3 [34]	61.60%	Cyst	0.645	0.826	0.724	0.465
		Normal	0.584	0.898	0.708	0.459
		Stone	0.568	0.462	0.509	0.412
		Tumour	0.76	0.295	0.425	0.566
Resnet50 [34]	73.80%	Cyst	0.735	0.641	0.685	0.625
		Normal	0.77	0.79	0.78	0.684
		Stone	0.745	0.692	0.717	0.706
		Tumour	0.706	0.827	0.762	0.673
Deep CNN [36]	99.25%	Cyst	0.97	1	0.98	1
		Normal	1	1	1	0.994
		Stone	1	0.99	1	0.988
		Tumour	1	0.98	0.99	1
Lightweight CNN [37]	99.52%	Cyst	0.994	0.999	0.998	0.995
		Normal	0.995	0.997	0.997	0.993
		Stone	0.997	0.979	0.988	0.986
		Tumour	0.993	0.995	0.995	0.993
Proposed method	99.89%	Cyst	0.999	1	1	0.999
		Normal	1	0.999	1	0.999
		Stone	0.984	0.997	0.991	0.990
		Tumour	1	0.996	0.998	0.998

## Data Availability

A publicly available dataset was used in this study, which can be found here: https://www.kaggle.com/datasets/nazmul0087/ct-kidney-dataset-normal-cyst-tumor-and-stone (accessed on 27 November 2019).
